# Supplementary material for: A cfDNA methylation-based tissue-of-origin classifier for cancers of unknown primary
Source: Nat Commun. 2024 Apr 17;15:3292. doi: 10.1038/s41467-024-47195-7 (PMC11024142; doi:10.1038/s41467-024-47195-7)
Supplement: Supplementary file 1 — Supplementary Information [file 41467_2024_47195_MOESM1_ESM.pdf]

Supplementary Information for:

CUPiD: A cfDNA methylation-based tissue-of-origin classifier for Cancers of Unknown Primary

Corresponding author: dominic.rothwell@cruk.Manchester.ac.uk

Supplementary Figures 1-6

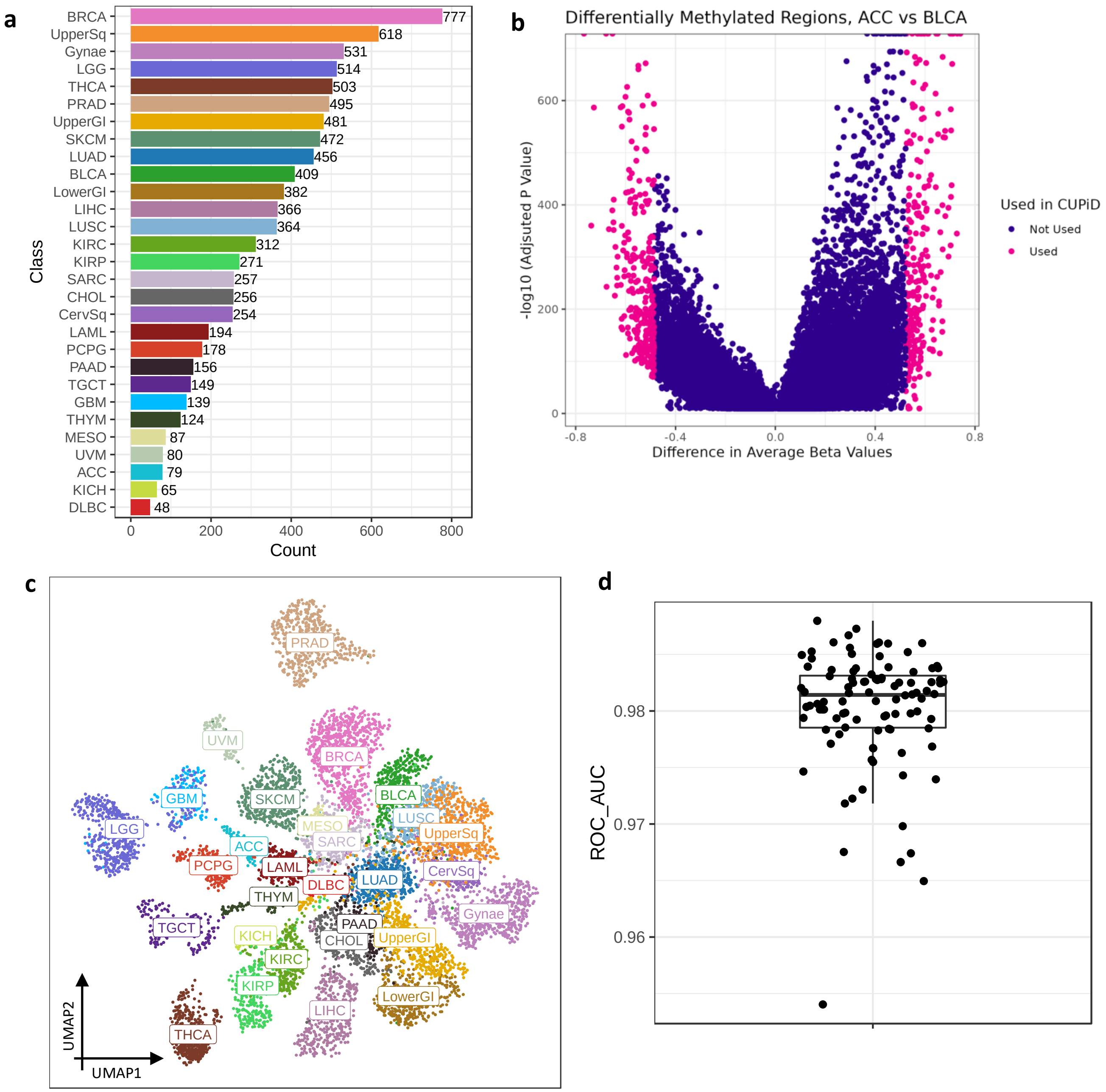

Supplementary Figure 1: CUPiD classifier development. **a** Number of arrays used in each cancer class. **b** Example volcano plot: difference in beta values against false discovery rate adjusted p-values (negative log scale) for the 59,918 DMRs between 79 ACC and 409 BLCA converted arrays. Highlighted in pink: top and bottom 250 regions with greatest magnitude of difference in beta values between each class selected to build the classifiers. **c** Two-dimensional Uniform Manifold Approximation and Projection for Dimension Reduction (UMAP) using all 234,979 regions of 9,017 converted arrays, showing separation of tumour classes. Class labels are superimposed over class centroids. **d** Multi-class Area Under the Receiver Operator Characteristics (AUROC) values for the 100 individual classifiers, evaluated on 10,611-11,508 held-out mixture samples per classifier. Boxes mark the 25th percentile (bottom), median (central bar) and 75th percentile (top); whiskers extend to 1.5 times the interquartile range. Class abbreviations are defined in Table 1. Source Data are provided as a Source Data file.

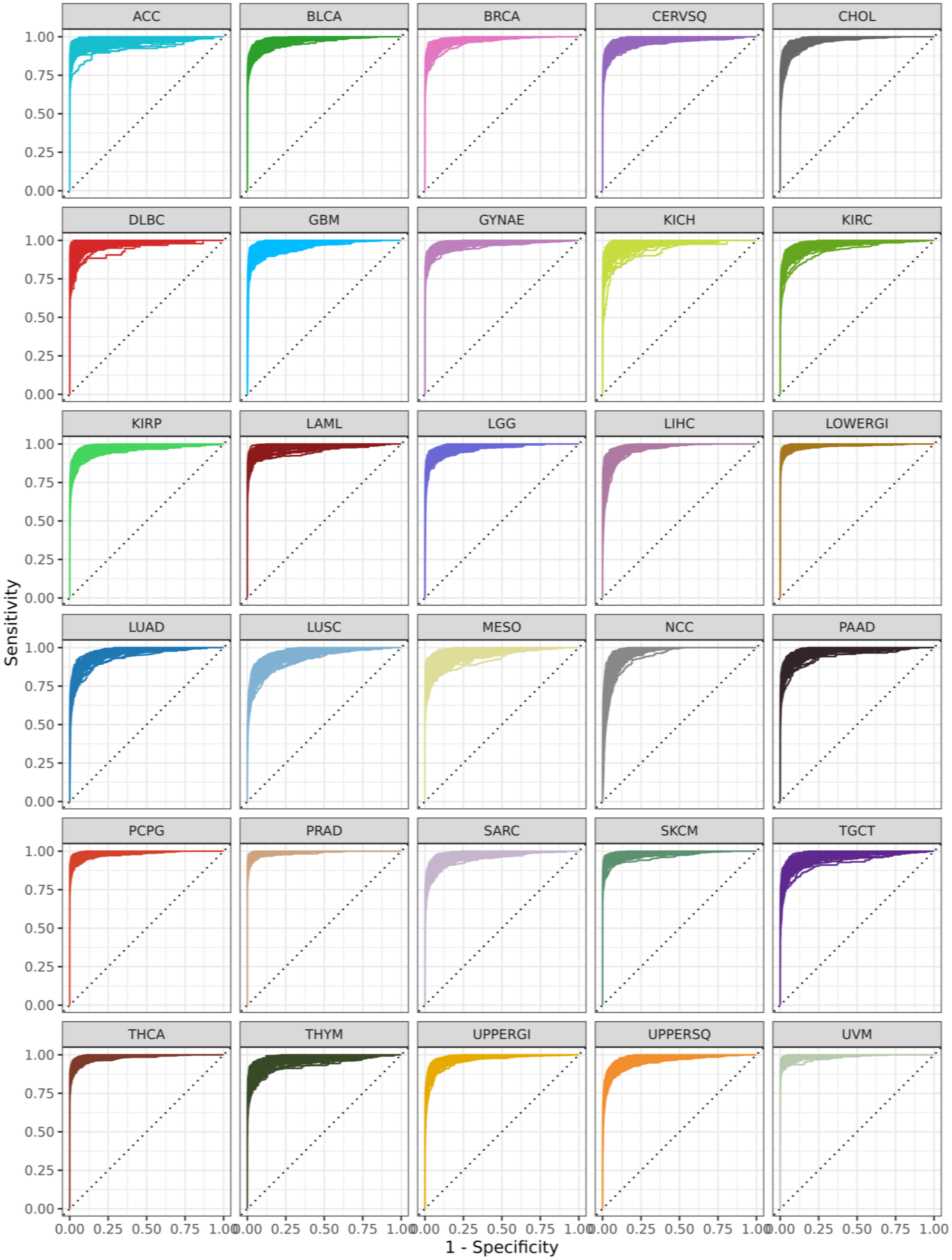

Supplementary Figure 2: CUPiD classifier performance. Individual Receiver Operating characteristic (ROC) curves for the 100 sub-classifiers, split by class. Evaluated on 10,611-11,508 held-out mixture samples per classifier. Class abbreviations are defined in Table 1. Source Data are provided as a Source Data file.

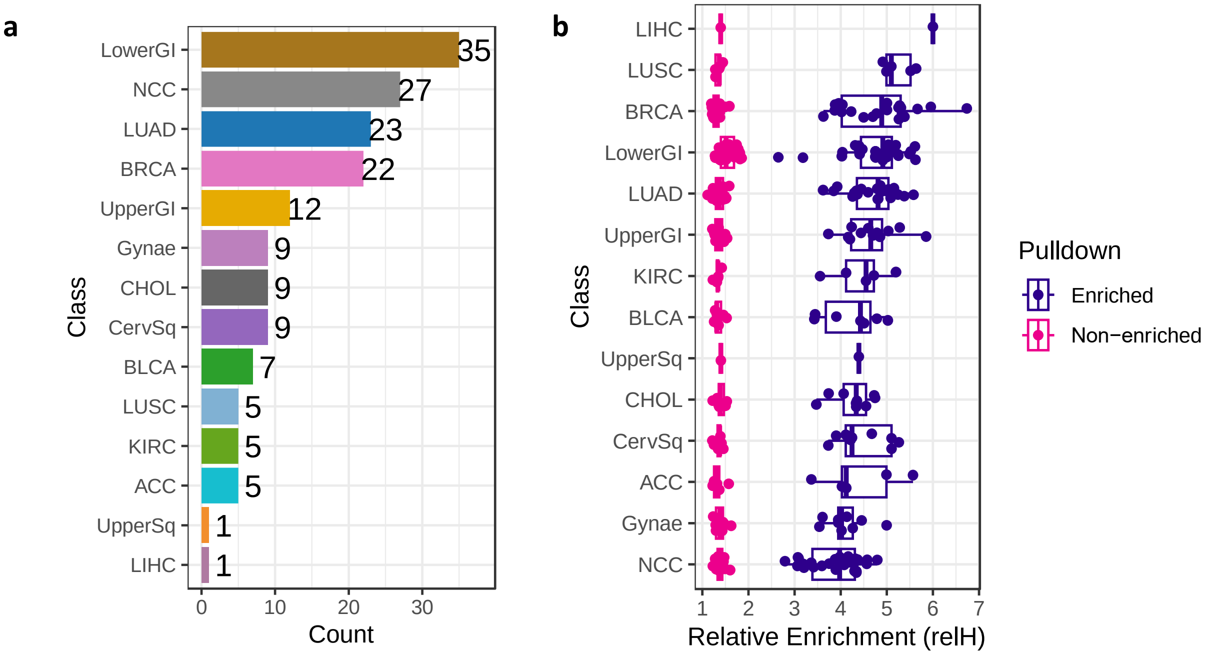

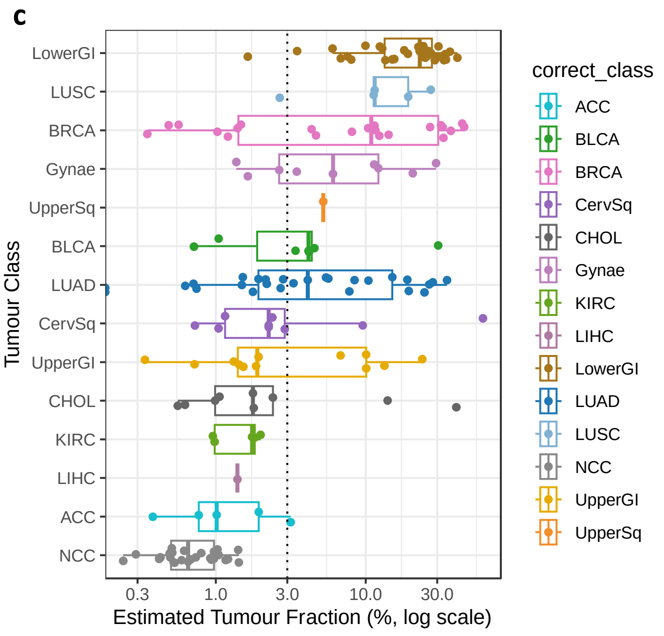

d

CUPiD concordance	CNA detected	No CNA detected	Total
Correct	84	37	121
No Prediction	2	16	18
Incorrect	3	1	4

Supplementary Figure 3: CUPiD testing in cancer and non-cancer cfDNA samples. a Number of T7-MBD-Seq samples of each class in the independent cfDNA test cohort. **b** Relative enrichment score (relH) of methylation enriched vs. Non-enriched fractions across the 170 cfDNA samples in test cohort, split by class. **c** Tumour Fraction (TF) estimated by ichorCNA using non-enriched fraction shallow whole genome sequencing on 170 cfDNA samples, split by class. 3% limit of detection cut-off shown. **d** Performance of CUPiD on 143 cfDNA samples from cancer patients split by copy number alteration (CNA) detection (by ichorCNA; defined as an estimated TF >3%). In B and C, boxes mark the 25th percentile (bottom), median (central bar) and 75th percentile (top); whiskers extend to 1.5 times the interquartile range. Cancer class abbreviations are defined in Table 1. Source Data are provided as a Source Data file.

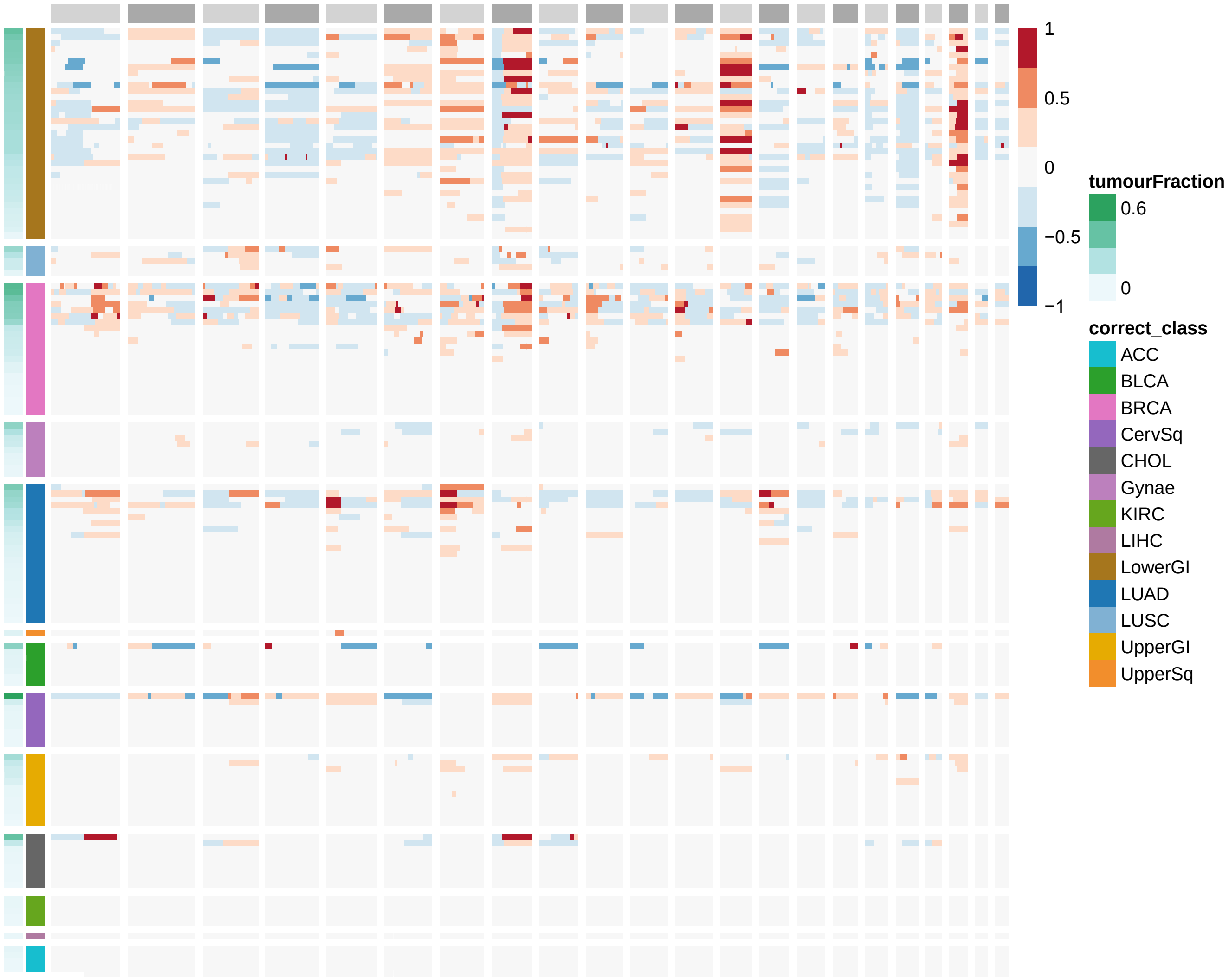

Supplementary Figure 4: Copy Number Analysis in test cohort. Copy Number Analysis (CNA) plot for 143 cfDNA samples from the independent test cohort as determined from shallow whole genome sequencing. Annotated by tumour class and estimated Tumour Fraction (TF) from ichorCNA. Red=gains, blue=losses. Class abbreviations are defined in Table 1. Source Data are provided as a Source Data file.

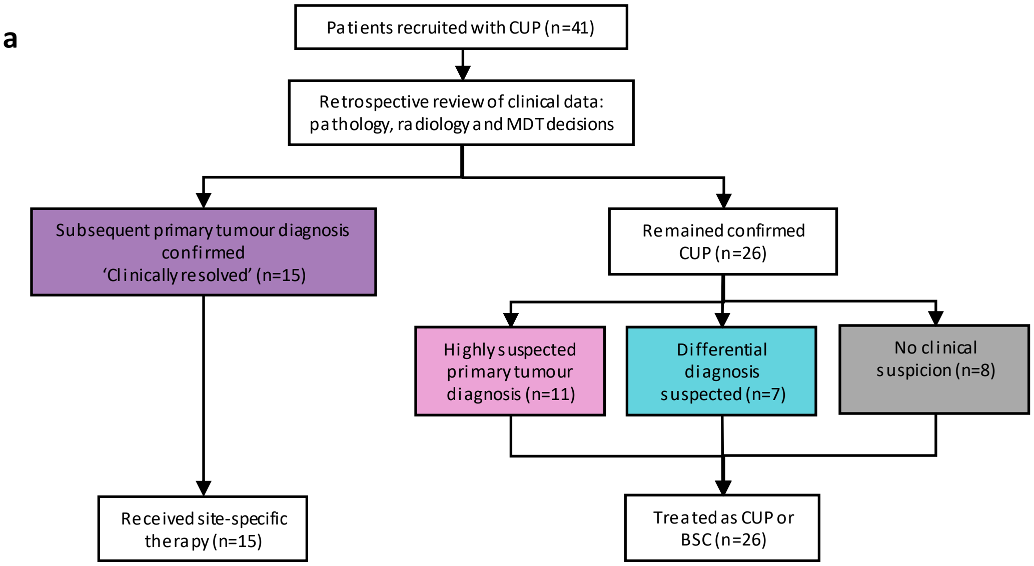

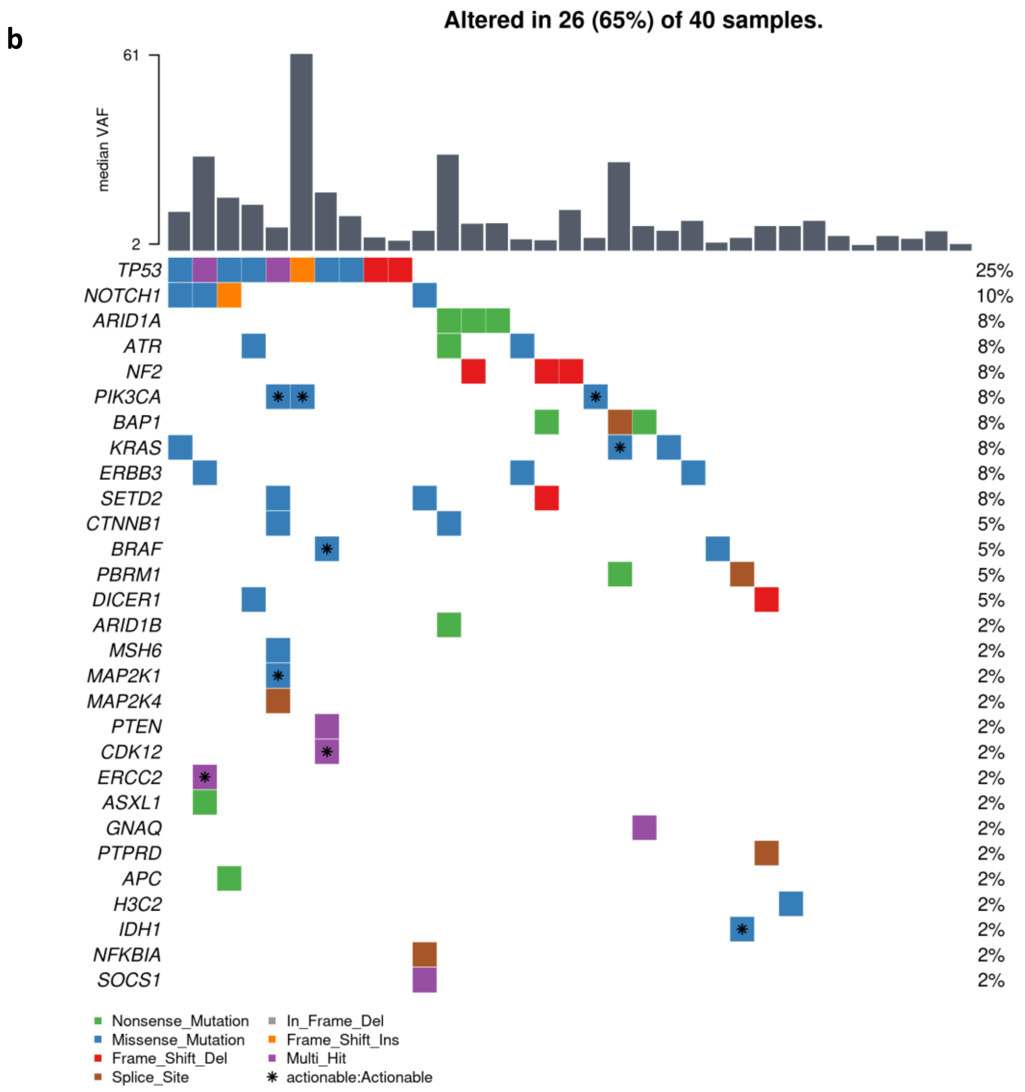

Supplementary Figure 5: CUP cohort summary and mutation data. a Flow diagram of CUP cohort diagnosis classification after retrospective review of clinical data (BSC = Best Supportive Care; MDT=Multi-disciplinary Team). **b** cfDNA mutational profiling with 641 gene targeted panel compared with matched germlines for 40 patients with CUP. Oncoplot shows alterations categorised as Oncogenic by oncoKB, actionable mutations highlighted by inset stars. Top panel: median variant allele frequency (VAF) of all alterations per patient. Source Data are provided as a Source Data file.

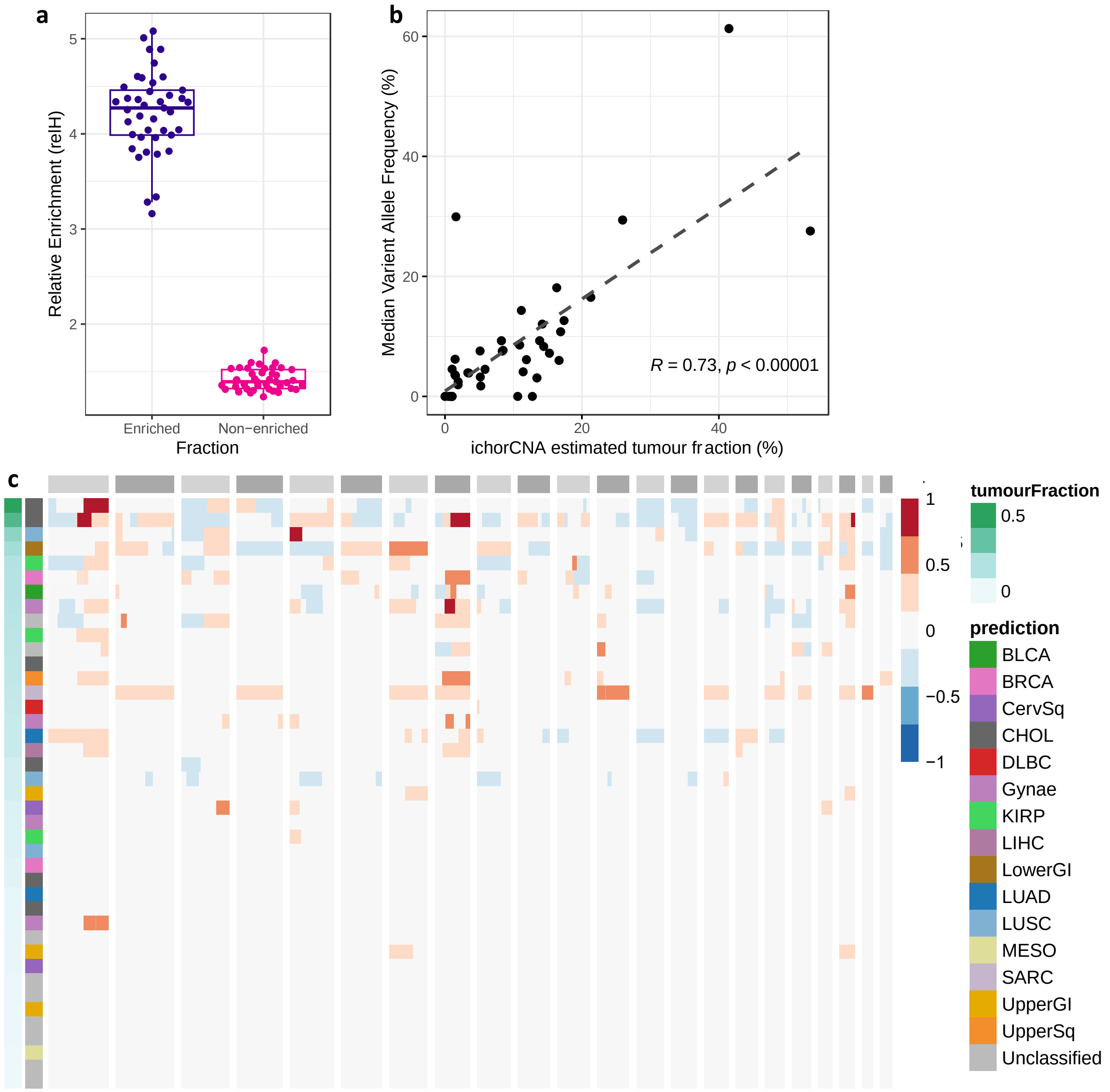

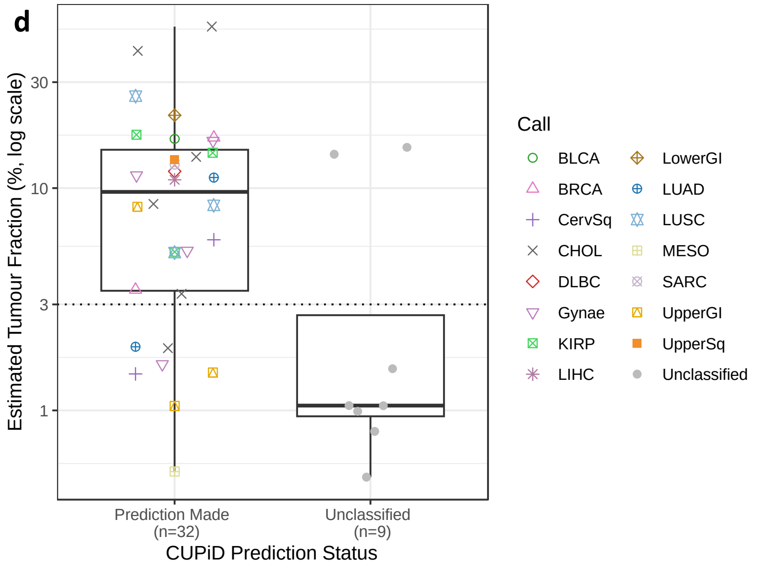

Supplementary Figure 6: cfDNA Methylation and CUPiD results in CUP cohort. a Relative enrichment score (relH) of methylation enriched vs. non-enriched fractions for 41 cfDNA samples from patients with CUP. **b** Correlation between tumour fraction estimated from ichorCNA against the median Variant Allele Frequency (VAF) from cfDNA mutation profiling with 641 gene panel for 40 patients with CUP. Pearson correlation (R value) and two-sided P value are shown. Dashed line shows linear regression fit. **c** CNA plot for 41 cfDNA samples from patients with CUP as determined from shallow whole genome sequencing. Annotated by CUPiD prediction and estimated TF from ichorCNA. Red=gains, blue=losses. Class abbreviations are defined in Table 1. **d** Estimated TF (from ichorCNA) of 41 cfDNA samples from patients with CUP grouped by CUPiD prediction status and coloured by predicted class. Boxes mark the 25th percentile (bottom), median (central bar) and 75th percentile (top); whiskers extend to 1.5 times the interquartile range. Dotted line denotes 3% tumour fraction. Source Data are provided as a Source Data file.
