# Supplementary material for: A cfDNA methylation-based tissue-of-origin classifier for cancers of unknown primary
Source: Nat Commun. 2024 Apr 17;15:3292. doi: 10.1038/s41467-024-47195-7 (PMC11024142; doi:10.1038/s41467-024-47195-7)
Supplement: Supplementary file 3 — Reporting Summary [file 41467_2024_47195_MOESM3_ESM.pdf]

nature portfolio

Corresponding author(s): Dominic Rothwell

Last updated by author(s): 23-02-2024

Reporting Summary

Nature Portfolio wishes to improve the reproducibility of the work that we publish. This form provides structure for consistency and transparency in reporting. For further information on Nature Portfolio policies, see our Editorial Policies and the Editorial Policy Checklist.

Statistics

For all statistical analyses, confirm that the following items are present in the figure legend, table legend, main text, or Methods section.

n/a	Confirmed	
☐	☒	The exact sample size (n) for each experimental group/condition, given as a discrete number and unit of measurement
☐	☒	A statement on whether measurements were taken from distinct samples or whether the same sample was measured repeatedly
☐	☒	The statistical test(s) used AND whether they are one- or two-sided<br>*Only common tests should be described solely by name; describe more complex techniques in the Methods section.*
☒	☐	A description of all covariates tested
☐	☒	A description of any assumptions or corrections, such as tests of normality and adjustment for multiple comparisons
☐	☒	A full description of the statistical parameters including central tendency (e.g. means) or other basic estimates (e.g. regression coefficient) AND variation (e.g. standard deviation) or associated estimates of uncertainty (e.g. confidence intervals)
☐	☒	For null hypothesis testing, the test statistic (e.g. F, t, r) with confidence intervals, effect sizes, degrees of freedom and P value noted<br>*Give P values as exact values whenever suitable.*
☒	☐	For Bayesian analysis, information on the choice of priors and Markov chain Monte Carlo settings
☒	☐	For hierarchical and complex designs, identification of the appropriate level for tests and full reporting of outcomes
☐	☒	Estimates of effect sizes (e.g. Cohen's d, Pearson's r), indicating how they were calculated

Our web collection on statistics for biologists contains articles on many of the points above.

Software and code

Policy information about availability of computer code

Data collection	Clinical data collection was perfomred using Microsoft Excel
Data analysis	The following tools were used in this publication:<br>BWA (0.7.17)<br>samtools (v1.15.1)<br>umi-tools (v1.1.2)<br>fastQC (v.0.11.9)<br>qualimap (v2.2.2d)<br>multiQC (v1.13)<br>cutadapt (v3.4)<br>NGSCheckmate (v1.0.0)<br>R (v4.2.0)<br>RStudioWorkbench (v1.4.1717-3)<br>NextFlow (v22.04.5)<br>QIAGEN CLC Genomics Workbench (v20.0.2 build 200002)<br>GATK (v4.2.5.0)<br>VEP (v193.1)<br>oncoKB (v3.17)<br>vcf2maf (v1.6.21)<br>gnomAD (v2.1.1)

R packages:
BiocParallel (v1.30.3)
BSgenome.Hsapiens.NCBI.GRCh38 (v1.3.1000)
butcher (v0.3.1)
dplyr(v1.0.0)
GEOquery (v2.66.0)
ggbeeswarm (v0.7.1)
ggplot2 (v3.4.1)
ggpubr (v0.4.0)
glue (v1.6.2)
hmmcopy (v1.32)
ichorCNA (v0.3.2)
janitor (v2.1.0)
maftools (v2.14.0)
mesa (v0.2.1, v0.2.2),
parsnip (v.1.0.0)
pheatmap (v1.0.12)
plyranges (v1.16.0)
purrr (v1.0.1)
qsea (v1.22.0)
readr (v2.1.4)
recipes(v1.0.3)
Rsamtools (v2.12.0)
stringr (v1.4.0)
swimplot (v1.2.0)
tibble (v3.1.8)
tidyr (v1.2.0)
uwot (v0.1.14)
vcfR (v1.13.0)
workflows (v1.1.0)
xgboost (v1.6.0.1)
yardstick (v1.0.0)

For manuscripts utilizing custom algorithms or software that are central to the research but not yet described in published literature, software must be made available to editors and reviewers. We strongly encourage code deposition in a community repository (e.g. GitHub). See the Nature Portfolio guidelines for submitting code & software for further information.

Data

Policy information about availability of data

All manuscripts must include a data availability statement. This statement should provide the following information, where applicable:

- Accession codes, unique identifiers, or web links for publicly available datasets
- A description of any restrictions on data availability
- For clinical datasets or third party data, please ensure that the statement adheres to our policy

The T7-MBD-seq and shallow whole genome sequencing data generated in this study have been deposited in the European Genome-Phenome Archive (EGA) under accession code EGAS00001007445 [https://ega-archive.org/studies/EGAS00001007445]. Pre-normalised TCGA data was downloaded from Xena Browser (https://tcga-pancan-atlas-hub.s3.us-east-1.amazonaws.com/download/jhu-usc.edu_PANCAN_HumanMethylation450.betaValue_whitelisted.tsv.synapse_download_5096262.xena.gz). Previously published cholangiocarcinoma methylation arrays were downloaded from the Gene Expression Omnibus under accession numbers GSE32079 [https://www.ncbi.nlm.nih.gov/geo/query/acc.cgi?acc=GSE32079], GSE49656 [https://www.ncbi.nlm.nih.gov/geo/query/acc.cgi?acc=GSE49656], GSE89803 [https://www.ncbi.nlm.nih.gov/geo/query/acc.cgi?acc=GSE89803]). Processed data (counts per 300bp window) for each sample is available upon request from Zenodo (http://doi.org/10.5281/zenodo.10678015). Data from applying the classifier to each sample is available from Zenodo (http://doi.org/10.5281/zenodo.10684337). Source data are provided with this paper, except the AUROC data which is available from Zenodo due to large file size (http://doi.org/10.5281/zenodo.10684337).

Research involving human participants, their data, or biological material

Policy information about studies with human participants or human data. See also policy information about sex, gender (identity/presentation), and sexual orientation and race, ethnicity and racism.

Reporting on sex and gender	Information on sex has been collected where provided and is available in Supplementary data files 2, 3 and 8. No Specific sex-effects were considered or controlled for, as CUP is a disease of both sexes. Sex was determined by self-reporting data collected at trial recruitment.
Reporting on race, ethnicity, or other socially relevant groupings	We have not reported race, ethnicity or other socially relevant groupings in this dataset and therefore this data has not been controlled for in analysis and its impact as a confounder and bias in classifier training and performance cannot be established.
Population characteristics	Age range and sex are provided for each individual within the study in Supplementary data 2, 3 and 8.<br>The non-cancer control cfDNA cohort used to train the classifier comprised 39 male and 40 female individuals with a median age of 63.<br>The independent test cohort comprised 60 male and 83 female patients with a median age of 56, as well as 14 male and 15

	female and 3 unspecified non-cancer controls with a median age of 65.<br>THe CUP cohort comprised 15 male and 26 female patients with a median age of 61.
Recruitment	All individuals in this study were recruited according to ethically approved protocols shown below. Selection bias may have been introduced as patients enrolled onto TARGET trial had all progressed on first or second line treatments. Patients with CUP recruited through the MCRC Biobank were treatment naive. All patients had stage IV cancer.
Ethics oversight	Patients with cancer were recruited through the TARGET (Tumour Characterisation to Guide Experimental Targeted Therapy) trial. Ethical approval obtained from the North-West (Preston) National Research Ethics Service in February 2015 (reference 15/NW/0078) and the trial was registered on the NIHR Central Portfolio Management System (reference CPMS ID 39172). Additional patients with CUP were recruited via the Manchester Cancer Research Centre (MCRC) Biobank CUP Project (application number 18_ALCO_01); ethically approved through the MCRC Biobank Research Tissue Bank Ethics (ref: 07/H1003/161+5, ethics code 18/NW/0092). All patients were recruited at The Christie NHS Foundation Trust, a UK tertiary cancer centre.<br>Non-cancer-control (NCC) samples were collected, with informed consent, from three sources: 1. The Community Lung Health Study (ethically approved study London - West London & GTAC Research Ethics Committee REC reference: 17/LO415); 2. The University of Manchester healthy normal volunteer study (University of Manchester Research Ethics Committee 4 (UREC4) University of Manchester ethics committee approval no. 2017-2761-4606); or 3. Purchased from Cambridge Bioscience (University of Manchester Research Ethics Committee ethics committee approval no. 2019-7920-11797).<br>All patients and individuals provided fully informed written consent and research was undertaken according to Good Clinical Practice guidelines and in accordance with declaration of Helsinki.

Note that full information on the approval of the study protocol must also be provided in the manuscript.

Field-specific reporting

Please select the one below that is the best fit for your research. If you are not sure, read the appropriate sections before making your selection.

☒ Life sciences ☐ Behavioural & social sciences ☐ Ecological, evolutionary & environmental sciences

For a reference copy of the document with all sections, see nature.com/documents/nr-reporting-summary-flat.pdf

Life sciences study design

All studies must disclose on these points even when the disclosure is negative.

Sample size	No statistical methods were used to predetermine sample size in this proof-of-concept study. All patients with CUP with plasma samples available from TARGET or the Manchester Biobank at the time of data generation were used. cfDNA samples from patients with known tumour types were selected based on the following conditions:<br>1. Tumour types and histological subtypes commonly suspected in CUP cohorts (Lung cancer, triple negative breast cancer, colorectal cancer, hepatobiliary, ovarian and renal carcinoma). In addition cervical, upper gastrointestinal, bladder, liver and adrenal carcinoma were selected where they had at least 5 patients available. They were chosen to better assess specificity across a broad range of cancer types.<br>2. All samples of the tumour types selected were processed where a minimum of 10ng of cfDNA was remaining for processing except for lung and colorectal cancer;<br>3. In cases of lung and colorectal cancer given the large numbers of patients available with these tumour types, samples were selected by those with the highest cfDNA yield/residual cfDNA for input into library preparation<br>Non-cancer-control samples collected under the Community Lung Health Study were selected if deemed to be cancer-negative by CT scan performed at the time of blood draw.
Data exclusions	Data was excluded if it did not fall within the inclusion parameters set out above.<br>In addition, data was excluded after processing if samples failed to meet the criteria below:<br>1. NGSCheckMate QC (n=2).<br>2. Relative methylation enrichment score (RelH) of more than 2.5 (n=5).<br>3. At least 40% of the hyperstable methylated regions were called by qsea with a beta value of 0.8 or above (n=2).<br>4. Found to be histological subtypes not present in the TCGA data (n=1, small cell histology).<br>5. Samples from patients where a second tumour diagnosis was suspected (n=1).
Replication	Individual clinical cfDNA samples were profiled only once due to limited available material. cfDNA samples from non-cancer and cancer individuals were used as independent test cohort and not used in the generation of the classifier itself. The performance of the classifier were not replicated in additional cfDNA samples since the aim of this study was to assess the feasibility of using cfDNA methylation profiling for determining tissue-of-origin of CUP. A further validation in a larger independent patient cohort will be performed.
Randomization	For classifier development, samples were randomised where necessary to ensure no overlap between training and test datasets. cfDNA samples used were limited to those available at the time of study and therefore exploratory. This study evaluated feasibility and proof-of-concept of the approach and therefore no randomisation applied or covariates controlled for. These will be applied to the design of a validation study.
Blinding	cfDNA samples used were limited to those available at the time of study and due to the exploratory nature of this study the investigators were not blinded to the tumour type of samples and patients in this proof-of-concept study. We expect blinding to be performed in a future validation of this classifier in a larger independent patient cohort.

Reporting for specific materials, systems and methods

We require information from authors about some types of materials, experimental systems and methods used in many studies. Here, indicate whether each material, system or method listed is relevant to your study. If you are not sure if a list item applies to your research, read the appropriate section before selecting a response.

Materials & experimental systems

n/a	Involved in the study
☒	☐ Antibodies
☒	☐ Eukaryotic cell lines
☒	☐ Palaeontology and archaeology
☒	☐ Animals and other organisms
☒	☐ Clinical data
☒	☐ Dual use research of concern
☒	☐ Plants

Methods

n/a	Involved in the study
☒	☐ ChIP-seq
☒	☐ Flow cytometry
☒	☐ MRI-based neuroimaging

Plants

Seed stocks	Not applicable
Novel plant genotypes	Not applicable
Authentication	Not applicable
