# Supplementary material for: A cfDNA methylation-based tissue-of-origin classifier for cancers of unknown primary
Source: Nat Commun. 2024 Apr 17;15:3292. doi: 10.1038/s41467-024-47195-7 (PMC11024142; doi:10.1038/s41467-024-47195-7)
Supplement: Supplementary file 4 — Description of Additional Supplementary Files [file 41467_2024_47195_MOESM4_ESM.pdf]

Description of Additional Supplementary Data Files

Supplementary Data 1

Description: Methylation array samples used for classifier generation or reasons for exclusion

Supplementary Data 2

Description: Clinical characteristics and T7-MBD-Seq metrics of 79 NCC cfDNA samples used for building the classifiers. Sequencing details for the enriched fraction (blue) and non-enriched fraction (green).

Supplementary Data 3

Description: Clinical characteristics and T7-MBD-Seq metrics of cfDNA from 143 patients and 27 NCCs. Sequencing details for the enriched fractions (blue) and non-enriched fractions (green).

Supplementary Data 4

Description: CUPiD predictions for the independent test cohort, mean values across the 100 sub-classifiers.

Supplementary Data 5

Description: Clinical and pathological characteristics of CUP cohort, tumour type enriched alterations (TTAEs) and CUPiD Predictions.

Supplementary Data 6

Description: Sequencing statistics for cfDNA and gDNA targeted mutation sequencing for the CUP cohort.

Supplementary Data 7

Description: List of relevant mutations found from cfDNA in the 40 patients with CUP with successful mutation profiling (*indicates nomenclature for Stop codon).

Supplementary Data 8

Description: Clinical characteristics and T7-MBD-Seq metrics from cfDNA samples of 41 patients with CUP. Sequencing details for the enriched fractions (blue) and non-enriched fractions (green).

Supplementary Data 9

Description: CUPiD Predictions in the CUP cohort
